# Wellington-bootstrap: differential DNase-seq footprinting identifies cell-type determining transcription factors

**DOI:** 10.1186/s12864-015-2081-4

**Published:** 2015-11-25

**Authors:** Jason Piper, Salam A. Assi, Pierre Cauchy, Christophe Ladroue, Peter N. Cockerill, Constanze Bonifer, Sascha Ott

**Affiliations:** Warwick Systems Biology Centre, University of Warwick, Coventry, CV4 7AL UK; Institute of Cancer and Genomic Sciences, College of Medical and Dental Sciences, Institute of Biomedical Research, University of Birmingham, Birmingham, B15 2TT UK; Department of Computer Science, University of Warwick, Coventry, CV4 7AL UK

**Keywords:** Transcriptional regulation, Transcription factors binding sites, Digital genomic footprinting, DNase-seq analysis, Gene regulatory networks

## Abstract

**Background:**

The analysis of differential gene expression is a fundamental tool to relate gene regulation with specific biological processes. Differential binding of transcription factors (TFs) can drive differential gene expression. While DNase-seq data can provide global snapshots of TF binding, tools for detecting differential binding from pairs of DNase-seq data sets are lacking.

**Results:**

In order to link expression changes with changes in TF binding we introduce the concept of differential footprinting alongside a computational tool. We demonstrate that differential footprinting is associated with differential gene expression and can be used to define cell types by their specific TF occupancy patterns.

**Conclusions:**

Our new tool, Wellington-bootstrap, will enable the detection of differential TF binding facilitating the study of gene regulatory systems.

**Electronic supplementary material:**

The online version of this article (doi:10.1186/s12864-015-2081-4) contains supplementary material, which is available to authorized users.

## Background

Digital DNaseI footprinting is a high throughput adaptation of classical DNaseI footprinting [1]. By subjecting nuclei to digestion by DNaseI, nucleosome-depleted genomic regions (accessible chromatin) that are sensitive to cleavage can be identified as DNase Hypersensitive Sites (DHSs) [2, 3]. Analyses of the patterns by which DNase I cuts within DHSs enables the identification of regions protected from digestion or “footprints”, which accurately demarcate transcription factor binding sites (TFBSs) at sub-30 bp resolution [4–10]. However, all currently available footprinting tools are designed for the analysis of a single DNase-seq data set at a time and thus will indiscriminately identify TFBSs that are part of a variety of different gene regulatory networks, limiting the ability to link regulatory events to cell- and tissue-specific processes, such as changes in cell fate or response to extracellular signals. For gene expression studies, a plethora of computational methods have been developed in order to identify genes that are differentially expressed in different conditions, thereby linking gene expression to changes in cellular status. However, a similar methodology that identifies differential transcription factor occupancy between DNase-seq datasets has so far been lacking, and methods such as DiffBind [11], designed for ChIP-seq are not appropriate for DNase-seq data. Here we describe the development of a novel computational tool to identify differential footprints (DFPs). We show that this tool can be used to link differential TF occupancy with differential gene expression and to identify closely related cell types by virtue of their TF occupancy patterns.

## Results and discussion

We have developed a conceptually simple and computationally efficient method, *Wellington-bootstrap*, for pairwise analysis of DNase-seq data sets. Wellington-boostrap builds on the Wellington method for detecting footprints in individual data sets [8]. Wellington uses knowledge of the strand imbalance around the TFBS introduced by the size-selection step in the double-hit DNase-seq method [12] in order to accurately detect footprints. This strand imbalance results in a characteristic pattern of reads aligning to the positive reference strand directly upstream of the TFBS and reads aligning to the negative reference strand directly downstream of the TFBS. With *Wellington-bootstrap*, footprints in data set *A* are detected and at each footprint locus a statistical test is performed testing whether pooling the data of data set *B* with *A* contributes to the footprint pattern or not. This yields a set of sites that are over-footprinted in *A* (under-footprinted in *B*) and associated DFP scores. Repeating the analysis with reversed roles for *A* and *B* yields over-footprinted sites in *B* (under-footprinted in *A*). We chose the approach of pooling data at individual loci in order to avoid biases that may be brought about by variations in sequencing depth.

**Fig. 1:**
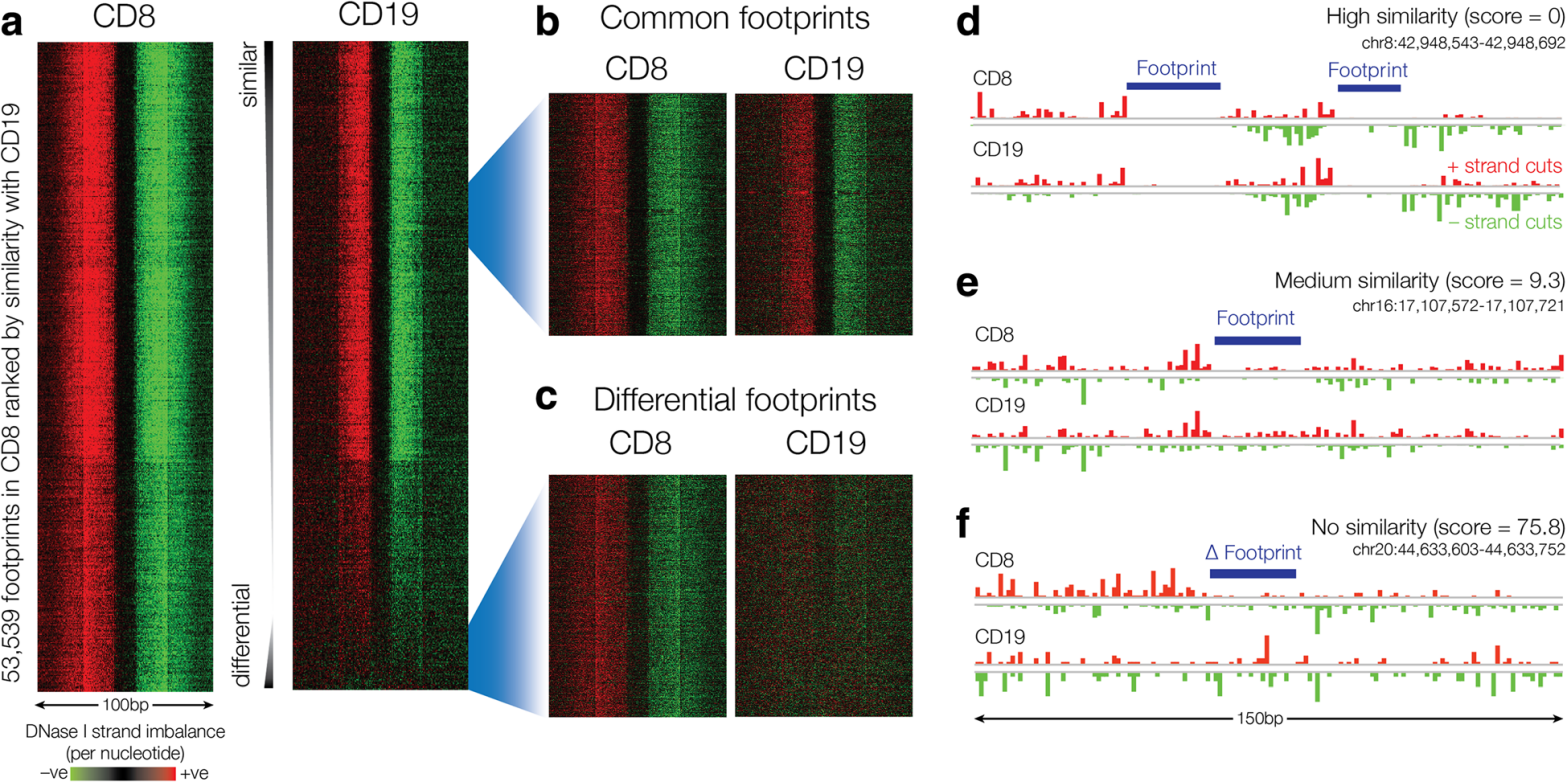
Wellington-bootstrap scores differential footprint occupancy between DNase-seq datasets. Wellington-bootstrap was applied at footprint loci in CD8+ cells to detect over-footprinted sites relative to CD19+ cells. **a** 53,539 loci were sorted by increasing Wellington-bootstrap score comparing CD8 vs CD19. Eight thousand seven hundred eighty loci were deemed to be DFPs. Red indicates an excess of positive strand cuts over negative strand cuts per nucleotide position, and green indicates an excess of negative strand cuts. Common footprints at the top of the heatmap share similar DNase activity as exemplified in (**b**) and (**d**) whereas footprints with increasing differential score towards the bottom of the heatmap show increasingly differential footprints (**c**, **e**, **f**)

Applying Wellington-bootstrap to publically available DNase-seq data for CD8+ and CD19+ cells we find 37,488 sites with evidence for DFPs. Furthermore, the Wellington-bootstrap score provides a way to order DFPs by the extent of footprint differences (Fig. 1). We found similar results making pairwise comparisons for all DNase-seq data sets for seven cell types from clinical tissue samples. A large proportion (up to 98.5 %, 43.9 % on average) of DFPs are found in DHSs that are shared between cell types, in particular in closely related cell types, indicating that these differences would be missed by restricting analyses to the presence or absence of DHSs (Table 1).

**Table 1:** A large proportion of differential footprints occurs in shared DHSs

Cell type A	Cell type B	DHSs in A	DHSs in B	DHSs shared between A and B	Sites over-footprinted in A	Sites in common DHSs over-footprinted in A	Sites over-footprinted in B	Sites in common DHSs over-footprinted in B
CD4	CD8	84,830	60,890	49,365	14,772	10,600 (71.8)	3874	3584 (92.5)
CD4	CD14	84,830	109,647	47,887	14,819	6219 (42)	17,932	7663 (42.7)
CD4	CD19	84,830	89,660	43,282	18,525	10,423 (56.3)	19,439	13,018 (67)
CD4	CD56	84,830	69,966	54,739	17,745	14,611 (82.3)	2616	2526 (96.6)
CD4	Spinal cord	84,830	197,751	34,812	24,652	9158 (37.1)	93,152	10,233 (11)
CD4	Fibroblasts	84,830	193,546	40,240	21,473	7087 (33)	118,265	11,741 (9.9)
CD8	CD14	60,890	109,647	32,185	11,602	6529 (56.3)	55,650	12,546 (22.5)
CD8	CD19	60,890	89,660	32,350	8780	5520 (62.9)	28,708	15,549 (54.2)
CD8	CD56	60,890	69,966	51,965	1458	1428 (97.9)	335	330 (98.5)
CD8	Spinal cord	60,890	197,751	27,631	13,128	5444 (41.5)	110,950	11,330 (10.2)
CD8	Fibroblasts	60,890	193,546	30,237	13,734	5894 (42.9)	156,418	15,573 (10)
CD14	CD19	109,647	89,660	36,349	48,031	15,909 (33.1)	27,111	18,140 (66.9)
CD14	CD56	109,647	69,966	33,900	54,850	17,845 (32.5)	7842	5357 (68.3)
CD14	Spinal cord	109,647	197,751	33,141	53,731	13,584 (25.3)	96,856	13,563 (14)
CD14	Fibroblasts	109,647	193,546	45,179	37,641	8383 (22.3)	108,482	12,677 (11.7)
CD19	CD56	89,660	69,966	35,766	31,561	19,315 (61.2)	5553	4130 (74.4)
CD19	Spinal cord	89,660	197,751	31,858	28,993	13,118 (45.2)	97,388	14,826 (15.2)
CD19	Fibroblasts	89,660	193,546	30,831	32,531	13,760 (42.3)	138,301	20,224 (14.6)
CD56	Spinal cord	69,966	197,751	28,731	8633	4404 (51)	110,996	13,892 (12.5)
CD56	Fibroblasts	69,966	193,546	31,469	9237	4769 (51.6)	154,923	20,024 (12.9)
Spinal cord	Fibroblasts	197,751	193,546	64,733	24,756	5497 (22.2)	35,202	9461 (26.9)

Number of DHSs and shared DHSs, number of over-footprinted sites, and number of over-footprinted sites located in the overlap of shared DHSs are shown for pairs of cell types. For closely related cell types most differential footprints tend to be found in common DHSs (e.g. CD4+ vs. CD56+). Developmentally distant cell types, however, often have a large number of DHSs that are cell type specific, and therefore the majority of differential footprints are in cell-type specific DHSs (e.g. CD56+ cells vs. fibroblasts)

**Fig. 2:**
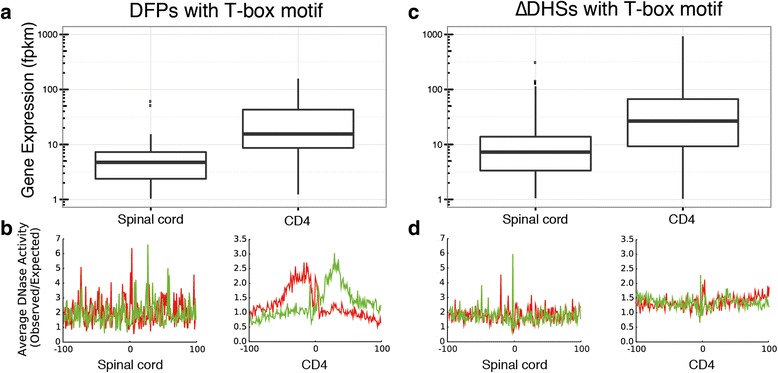
Differential footprints reveal links between TF binding and gene expression. **a** Differential gene expression (p < 0.005, Mann–Whitney *U* test) of all genes that have a differential CD4 footprint containing a match for the T-box motif in their promoter. **b** Average bias-corrected DNase-seq cleavage profiles (red: positive strand cuts, green: negative strand cuts) centred on T-box motifs in promoters of genes from (**a**) show evidence for binding of T-box motifs in CD4+ cells, but not in spinal cord cells. Genes over-footprinted for T-box in CD4+ cells are also over-expressed, confirming a known lineage-determining link. **c** Differential gene expression of all genes that have a differential CD4+ DHS containing a match for the T-box motif in their promoter. **d** Average bias-corrected DNase-seq cleavage profiles centred on T-box motifs in promoters of genes from (**c**) do not show evidence for binding in either cell type. The differential expression observed in (**c**) cannot be linked to TF binding using differential DHS scores alone

Using Spinal cord and CD4+ cells as example we tested the ability of DFPs to re-discover known regulatory links and predict gene expression. In CD4+ cells, the T cell specific TF T-bet binds T-box motifs and enhances target gene expression as part of the Th1-differentiation programme [13]. In spinal cord cells, the TF MAZ is known to be involved in neuronal development [14]. Among the set of all DFPs located near transcriptional start sites and over-footprinted in CD4+ cells we identified the sites containing a match for the T-box motif. We found that the expression of nearby genes differed significantly, with the DNase-seq data providing strong evidence for the presence of protein binding in CD4+ cells and absence of binding in spinal cord cells (Fig. 2a, b). Similarly, we found that a link between binding to MAZ motifs and gene expression was evident (Additional file 1: Figure S1a, b), demonstrating the ability of the DFP approach to isolate the effect of individual TFs from their genomic context.

Previously, comparisons of total read numbers in DHSs have been used as a means of analysing pairs of DNase-seq data sets [15]. We identified the set of T-box motif-containing DHSs in gene promoters with the highest increase in read numbers in CD4+ cells compared to spinal cord cells. While these showed differential expression of nearby genes, no evidence for differences in binding was revealed using this approach (Fig. 2c, d). Similarly, this approach did not reveal the regulatory link between MAZ binding and target gene expression (Additional file 1: Figure S1c, d). The cleavage profiles shown in Fig. 2b, d and Additional file 1: Figure S1b, d have been corrected for the known sequence preference of the DNaseI enzyme. Additional file 1: Figure S2 compares cleavage profiles with and without this correction. Overall, this suggests that unlike DFPs, motif analysis of DHSs is insufficient to link a given TF to changes in gene expression, making the use of DFPs a valuable tool for this purpose.

**Fig. 3:**
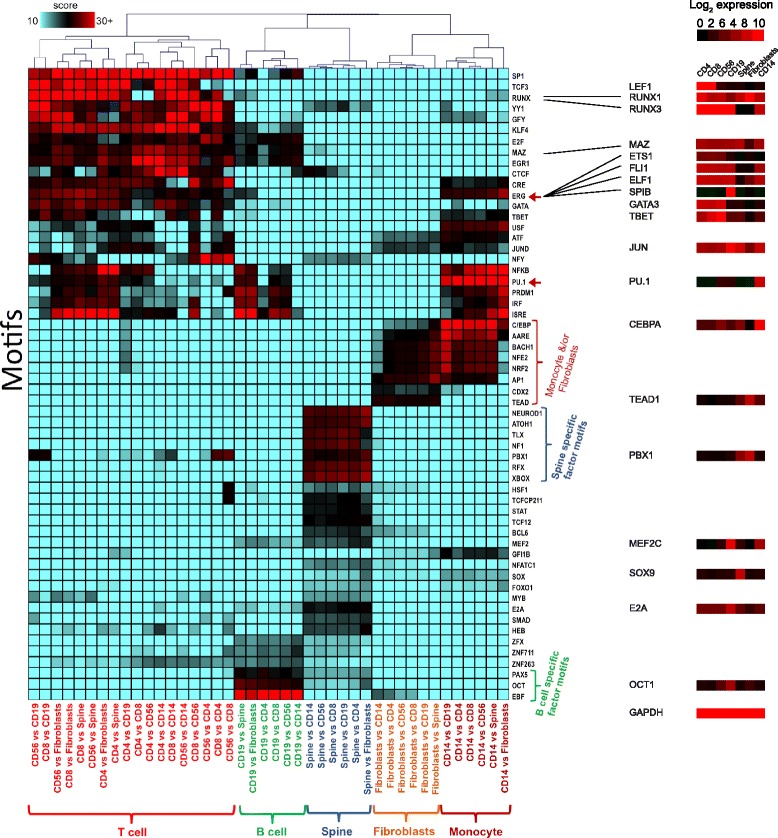
Analysis of differential footprints in the haematopoietic system reveals cell-type specific transcription factor networks. Differential footprints in 42 pairs of cell types and matches to known motifs inside differential footprints were determined using DNase-seq data from the NIH Roadmap Epigenomics project. Coloured boxes represent motif frequency with red indicating higher than average frequency. Hierarchical clustering was applied to rows and columns. Red arrows highlight members of the ETS family of transcription factors. BioGPS gene expression of typical tissue-specific TFs corresponding to motifs enriched in DFPs is shown to the right, with GAPDH as a positive control (bottom). The result correctly groups cell types and reveals known and likely regulatory factors

We sought to further explore the potential of the DFP approach to reveal cell type-specific regulatory mechanisms. Using differential footprints amongst all pairs of DNase-seq data sets of seven primary cell types, we determined the relative frequency of motif occurrences for a set of known TF binding motifs and used this data to cluster the set of pairs of cell lines as well as the set of TF binding motifs (Fig. 3). This analysis generated a number of striking results. Firstly, our DFP methodology combined with clustering recovered the different cell types as separate clusters. Moreover, it was able to distinguish between the different cell types as their specifically occupied DNA sequences clustered together. Secondly, the analysis gave interesting insights into the relative role of individual TF families within a given cell type. For example, high differential C/EBP motif occupancy was a classifier for CD14+ monocytes as well as fibroblasts, both of which express CEBPA, but the relative motif frequency was lower in fibroblasts which agrees with the fact that this factor is absolutely essential for monocyte but not fibroblast development [16, 17]. Another interesting finding was that increased occupancy of PU.1 motifs was a classifier for both B cells and CD14+ monocytic cells where this factor plays an important role [18], but a significant number of such sites were occupied also in T cells. PU.1 is expressed in hematopoietic stem cells from which all hematopoietic cells originate, but its expression is down-regulated in T cells and its overexpression is detrimental for their development [19]. There is some overlap between the binding specificities of different ETS-family proteins [20]. It is therefore possible that some of these sequences are bound by another ETS factor in T cells. Importantly, gene expression patterns of typical TFs corresponding to motifs enriched in differential footprints showed tissue-specific expression, whereby they tended to be expressed in the cell type in which they were differentially footprinted. Comparable motifs could also be obtained in an unbiased way via *de novo* motif discovery, as exemplified for a CD19 versus CD4 differential footprinting analysis (Additional file 1: Figure S4). These motif results are supported by previous findings in B-cells [21, 22].

To facilitate the wide-spread use of our method, we provide an implementation of Wellington-bootstrap alongside a substantial update of pyDNase, including increased performance and parallelised computations. This is released as open source under the GPLv3 license at https://github.com/jpiper/pyDNase.

## Conclusions

In conclusion, we introduce a fundamental and useful method for differential footprints, provide a tool for the detection of DFPs, and reveal the potential of this approach to map regulators to context-specific gene expression. Applying this methodology will be highly relevant for classifying closely related cell types, both in the normal, but also the diseased state and to assess the relative importance of specific TF families for each state. Wellington-bootstrap is applicable to any pair of DNase-seq data sets obtained with comparable experimental protocols including perturbation and time course experiments, making it a widely applicable approach for the identification of transcriptional regulatory hierarchies.

## Methods

### DNase-seq data and peak-finding

DNase-seq data from the NIH Roadmap Epigenomics project [23] were downloaded from the Short Read Archive (accessions CD4: SRX214041, CD8: SRX204403, CD19: SRX342324, CD14: SRX252602, CD56: SRX204402, spinal cord: SRX121287, fibroblasts: SRX135564) and were aligned to hg19 using Bowtie 2.2.0 [24] using the default parameters. DNase hypersensitive site detection for all DNase-seq data was performed using HOMER’s findPeaks.pl tool [25] with the parameters “findPeaks -region -size 500 -minDist 50 -o auto -tbp 0”.

### Differential footprinting – Wellington-bootstrap

Wellington-bootstrap first determines Wellington footprints in the primary dataset. At each footprint locus the data from the comparator dataset is added and the Wellington footprint score for the pooled data evaluated. Wellington-bootstrap then assesses if the change in footprint score is a consequence of the increase in read numbers after pooling reads or if the data from the comparator dataset makes a contribution to the footprint structure. To do this, the comparator data is randomly shuffled 1000 times, pooled, and the Wellington footprint score evaluated (see example in Additional file 1: Figure S3). Shuffling is done in a strand independent manner, randomising the positions of the counts of 5′ DNase cuts per base pair on the positive and negative strand. The score of pooled data without shuffling is assessed against the bootstrap distribution and the percentile used as the differential footprinting score. Low scores indicate non-differential footprints, high scores differential footprints. Figure 1 shows that sorting by this score orders pairs of footprints in an intuitive manner enabling the user to retrieve the most differential footprints while choosing the stringency. 10 was used as the threshold in this work. The role of the two datasets is reversed and the computation repeated to obtain both over- and under-footprinted sites.

It was initially thought that flexibility would be required regarding the width of the footprint and its position in the two datasets. Whilst initial methods were developed to take this into consideration, we found that this provided no improvement to the method, yet yielded a significant speed decrease. This analysis has been implemented in the wellington_bootstrap.py script as part of pyDNase 0.2.0.

### Differential DHSs – Fig. 2 and Additional file 1: Figure S1

Differential DHSs (∆DHS) scores were calculated according to the method proposed by He et al. 2012 [15] and the implementation used here has been provided as dnase_dshs_scores.py in pyDNase 0.2.0. DHSs were then filtered to those that were within 2 kb of a single TSS using the hg19 UCSC knownGene gene model, and the DHSs showing the top and bottom *n* = 1000 ∆DHS scores were chosen as the differential DHSs. Equivalent results were obtained using the following alternative choices for *n*: 50 (matching the number of DFPs used in Fig. 2a,b), top 476 and bottom 300 (corresponding to two standard deviations difference to mean ∆DHS score), 1403 (corresponding to top and bottom 10 %).

### RNA-seq analysis

RNA-seq data were downloaded from the Short Read Archive (accessions CD4: SRR643766, spinal cord: SRR980477) and FPKM was estimated using Tophat 2.0.11 [26] and Cufflinks 2.1.1 [27] with the Illumina iGenomes UCSC hg19 knownGene GTF file.

### Motif analysis – Fig. 3

The annotatePeaks.pl script of the HOMER package was used to find occurrences of known motifs in peaks. Wellington-bootstrap was applied to compute 42 sets of differential footprints for all ordered pairs of the seven cell types used (CD4/CD8 T-cells, CD56 NK cells, CD19+ B cells, spinal cord cells, fibroblasts, CD14+ monocytes). To analyse motif frequencies in differential footprints motif search was done within the differential footprint coordinates extended by 10 bp either side. Relative motif frequencies were calculated as

$$ \mathrm{R}\mathrm{e}\mathrm{lative}\kern0.5em \mathrm{frequency}\kern0.5em \mathrm{motif}\kern0.5em i\kern0.5em \mathrm{in}\kern0.5em \mathrm{comparison}\kern0.5em j=\left({n}_{ij}/{M}_j\right)\times \left(C{\varSigma}_j{M}_j/{\varSigma}_j{n}_{ij}\right), $$

where *C* is a scaling constant, *n*_*ij*_ is the number of differential footprints in set *j (j = 1,2,…,42)* that are occupied by motif *i (i = 1, 2,….,I), I* is the total number of motifs used, and *M*_*j*_ the total number of differential footprints in each subset *j (j = 1,2,…,42).* A matrix was generated and motif scores displayed as a heatmap after hierarchical clustering with Euclidean distance and complete linkage. Blue indicates low relative frequency; red/black indicates high relative frequency. Heatmaps were generated using Mev of the TM4 microarray software suite [28].

### Gene expression of transcription factors in all tissues

HG_U133A microarray expression data from BioGPS [29], covering 84 normal tissues as well as penis foreskin fibroblasts (GEO accession number GSE4521) were retrieved, concatenated and normalized via R using the normalizeQuantiles function of limma [30]. Heatmap images were obtained via Java Treeview [31].

### De novo motif discovery

The findMotifsGenome.pl script of the HOMER package was used to perform *de novo* motif discovery in CD19 versus CD4 differential footprints.

### pyDNase 0.2.0 – cutting bias correction

In order to plot cut bias corrected average DNase cleavage plots, the DNaseI 6-mer cutting bias data from naked genomic data from the IMR90 cell line and for each region an ‘expected count’ was calculated using the ‘predicted count’ formula from He et al. 2014 [32]. The observed cuts at each base pairs were then divided by the expected counts. Bias correction modes have been added to the plotting scripts in pyDNase that can be invoked with the ‘-b < genome.fa>’ option. The BAMHandlerWithBias class in pyDNase provides underlying access to the bias correction for power users. In this we have provisioned the ability for the user to supply a Variant Call Format (VCF) file so that the reference DNA sequence can be corrected using SNPs present in the sample being analysed if desired.

### pyDNase 0.2.0 – other new features and improvements

pyDNase 0.2.0 represents a major release for pyDNase, bringing several improvements. The core Wellington algorithm was reimplemented in C, and the underlying code structure was refactored in order to allow for parallelisation of Wellington score calculation. On a dual 2.66Ghz i7 Xeon workstation with 8 cores, footprinting a single dataset takes approximately 30 min, compared to up to 20 h previously on a single core – this performance increase scales linearly with number of cores utilised. In addition, a number of analysis scripts have been added to the pyDNase library for calculating ∆DHS scores, calculating Wellington-bootstrap scores, annotation of BED files with Footprint Occupancy Scores, and the annotation of a BED file with DNase cuts. A comprehensive DNase-seq footprinting tutorial has also been added to assist those new to DNase-seq analysis and DNase-seq footprinting. Full details can be found at the pyDNase github repository (https://github.com/jpiper/pyDNase).

### Data access

All software is released as open source under the GPLv3 license at http://jpiper.github.io/pyDNase/.

## Additional file

Additional file 1: Figure S1. Differential footprints reveal links between TF binding and gene expression. (a) Differential gene expression (p < 0.005, Mann–Whitney *U* test) of all genes that have a differential spinal cord footprint containing a match for the MAZ motif in their promoter. (b) Average bias-corrected DNase-seq cleavage profiles (red: positive strand reads, green: negative strand reads) for MAZ sites in promoters of genes from (a) show evidence for binding of MAZ motifs in spinal cord cells, but not in CD4+ cells. Genes over-footprinted for MAZ in spinal cord cells are also over-expressed, confirming a known lineage-determining link. (c) Differential gene expression of all genes that have a differential spinal cord DHS containing a match for the MAZ motif in their promoter. (d) Average bias-corrected DNase-seq cleavage profiles for MAZ sites in promoters of genes from (c) show evidence for binding in both cell types. The differential expression observed in (c) cannot be linked to differences in TF binding using differential DHS scores alone. **Figure S2.** Bias correction refines profiles of average cutting. For T-box-containing loci of differential footprints used in Fig. 2b average DNaseI cleavage profiles are shown before (a, c) and after (b, d) correcting for the sequence specificity of DNaseI cleavage using a 6-mer model (He et al., 2013). Plots (b) and (d) are the ones shown in Fig. 2b. **Figure S3.** Example of a footprint deemed non-differential. (a) Red (green) bars represent numbers of 5′ ends of reads aligning to the positive (negative) reference strand. Vertical black lines indicate footprint region. (b) Bootstrap distribution for data shown in (a). Nucleotide positions in CD19 data were randomly shuffled and the distribution of Wellington footprint scores after pooling the shuffled CD19 data and the fibroblast data was determined. Blue vertical bar shows the Wellington score after pooling data without shuffling. Green: Wellington footprint score in fibroblast data. Red: footprint score in CD19 data. As pooling without shuffling yields a better footprint score than pooling with shuffling the footprint is considered non-differential. **Figure S4.** Example of de novo motif discovery in differential footprints. HOMER de novo motif discovery results in footprints differentially enriched in CD19 versus CD4 lymphocytes. The top 6 motifs are shown sorted by increasing p-value. (DOCX 545 kb)

## Abbreviations

DFP: Differential footprint
DHS: DNase hypersensitive site
∆DHS: Differential DNase hypersensitive site
TF: Transcription factor
TFBS: Transcription factor binding site
