# Supplementary material for: Wellington-bootstrap: differential DNase-seq footprinting identifies cell-type determining transcription factors
Source: BMC Genomics. 2015 Nov 25;16:1000. doi: 10.1186/s12864-015-2081-4 (PMC4658755; doi:10.1186/s12864-015-2081-4)
Supplement: Additional file 1: Figure S1. — Differential footprints reveal links between TF binding and gene expression. (a) Differential gene expression (p < 0.005, Mann–Whitney U test) of all genes that have a differential spinal cord footprint containing a match for the MAZ motif in their promoter. (b) Average bias-corrected DNase-seq cleavage profiles (red: positive strand reads, green: negative strand reads) for MAZ sites in promoters of genes from (a) show evidence for binding of MAZ motifs in spinal cord cells, but not in CD4+ cells. Genes over-footprinted for MAZ in spinal cord cells are also over-expressed, confirming a known lineage-determining link. (c) Differential gene expression of all genes that have a differential spinal cord DHS containing a match for the MAZ motif in their promoter. (d) Average bias-corrected DNase-seq cleavage profiles for MAZ sites in promoters of genes from (c) show evidence for binding in both cell types. The differential expression observed in (c) cannot be linked to differences in TF binding using differential DHS scores alone. Figure S2. Bias correction refines profiles of average cutting. For T-box-containing loci of differential footprints used in Fig. 2b average DNaseI cleavage profiles are shown before (a, c) and after (b, d) correcting for the sequence specificity of DNaseI cleavage using a 6-mer model (He et al., 2013). Plots (b) and (d) are the ones shown in Fig. 2b. Figure S3. Example of a footprint deemed non-differential. (a) Red (green) bars represent numbers of 5′ ends of reads aligning to the positive (negative) reference strand. Vertical black lines indicate footprint region. (b) Bootstrap distribution for data shown in (a). Nucleotide positions in CD19 data were randomly shuffled and the distribution of Wellington footprint scores after pooling the shuffled CD19 data and the fibroblast data was determined. Blue vertical bar shows the Wellington score after pooling data without shuffling. Green: Wellington footprint score in fibroblast data. Red: footpri [file 12864_2015_2081_MOESM1_ESM.docx]

**SUPPLEMENTAL FIGURES**

**Wellington-bootstrap: Differential DNase-seq footprinting identifies cell-type determining transcription factors**

Jason Piper, Salam A. Assi, Pierre Cauchy, Christophe Ladroue, Peter N. Cockerill, Constanze Bonifer, Sascha Ott

**
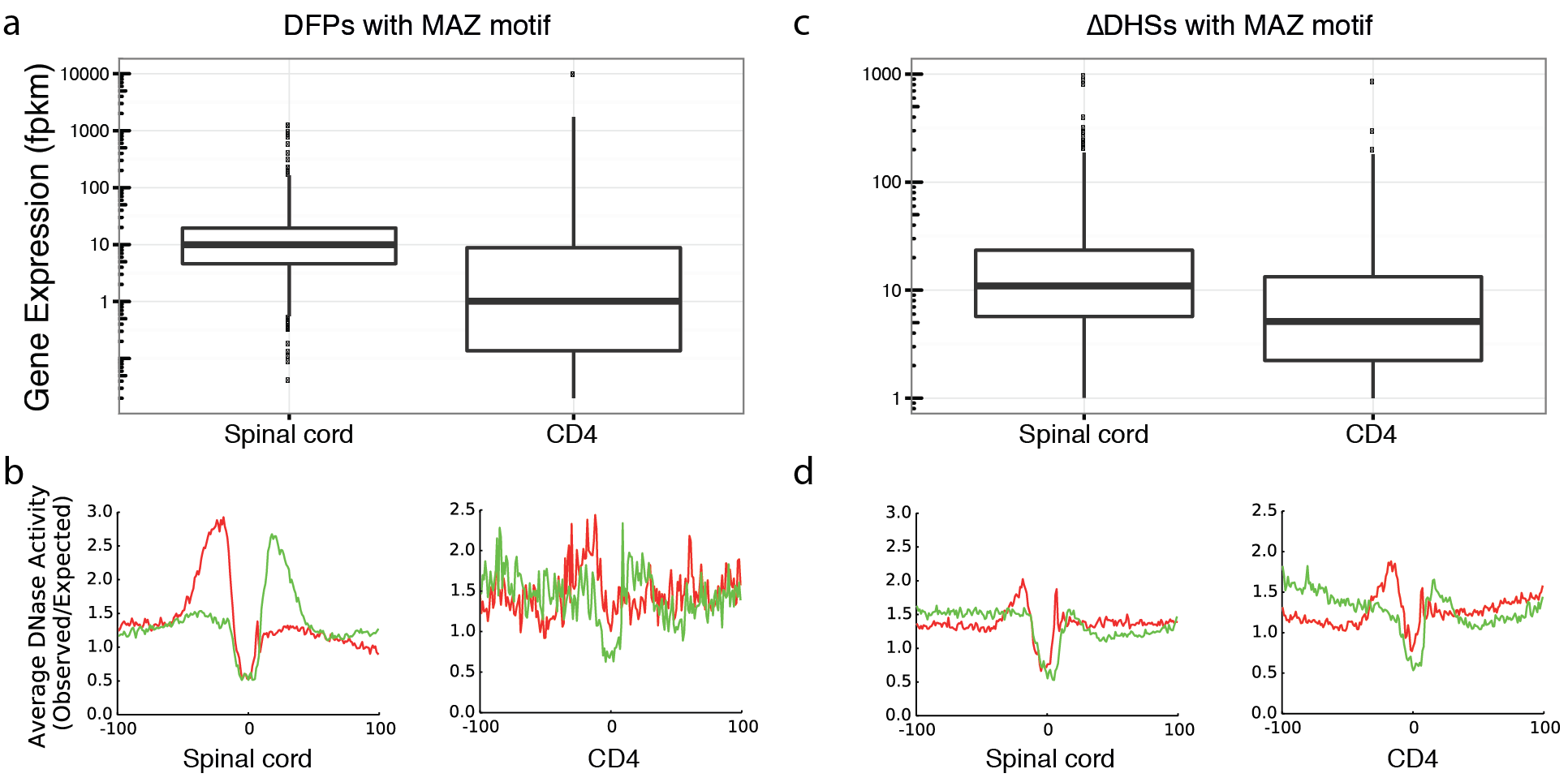
**

**Figure S1.** **Differential footprints reveal links between TF binding and gene expression**. (**a**) Differential gene expression (p<0.005, Mann-Whitney U test) of all genes that have a differential spinal cord footprint containing a match for the MAZ motif in their promoter. (**b**) Average bias-corrected DNase-seq cleavage profiles (red: positive strand reads, green: negative strand reads) for MAZ sites in promoters of genes from (**a**) show evidence for binding of MAZ motifs in spinal cord cells, but not in CD4+ cells. Genes over-footprinted for MAZ in spinal cord cells are also over-expressed, confirming a known lineage-determining link. (**c**) Differential gene expression of all genes that have a differential spinal cord DHS containing a match for the MAZ motif in their promoter. (**d**) Average bias-corrected DNase-seq cleavage profiles for MAZ sites in promoters of genes from (**c**) show evidence for binding in both cell types. The differential expression observed in (**c**) cannot be linked to differences in TF binding using differential DHS scores alone.

**
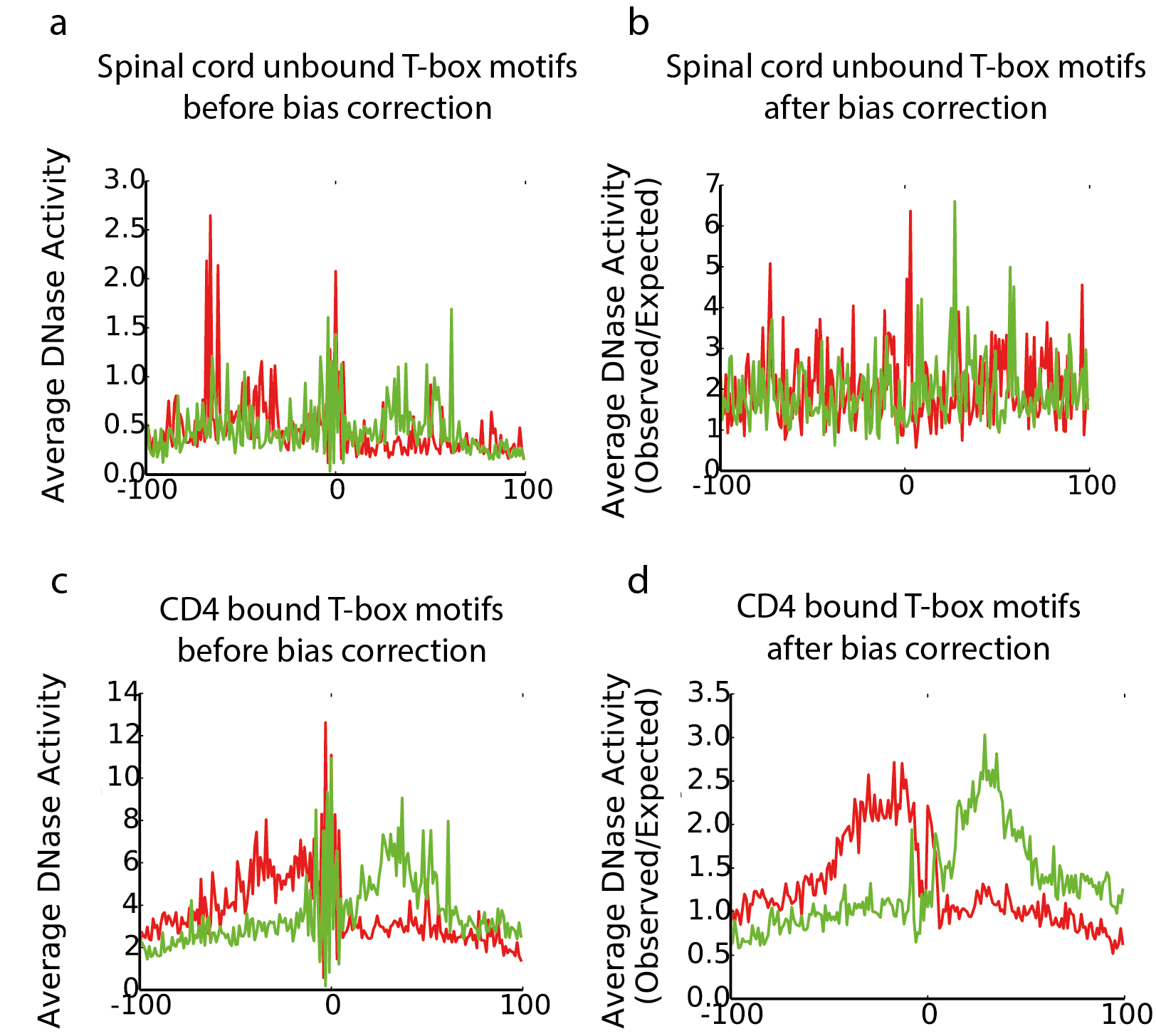
**

**Figure S2.** **Bias correction refines profiles of a­­­verage cutting.** For T-box-containing loci of differential footprints used in **Figure 2b** average DNaseI cleavage profiles are shown before (**a, c**) and after (**b, d**) correcting for the sequence specificity of DNaseI cleavage using a 6-mer model (He et al., 2013). Plots (**b**) and (**d**) are the ones shown in **Figure 2b**.

a

| 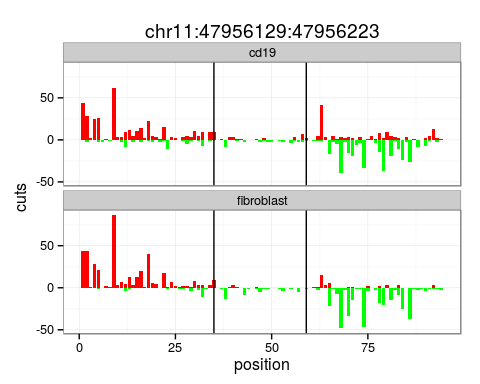  b |
| --- |
| 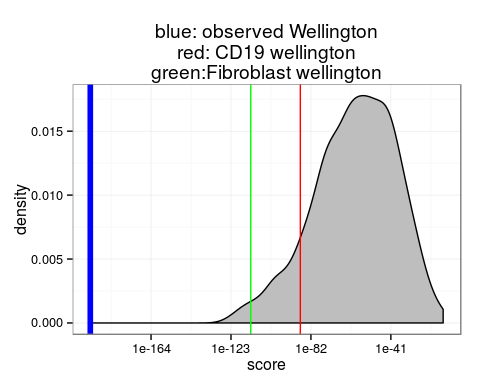 |

**Figure S3. Example of a footprint deemed non-differential.** (**a**) Red (green) bars represent numbers of 5’ ends of reads aligning to the positive (negative) reference strand. Vertical black lines indicate footprint region. (**b**) Bootstrap distribution for data shown in (**a**). Nucleotide positions in CD19 data were randomly shuffled and the distribution of Wellington footprint scores after pooling the shuffled CD19 data and the fibroblast data was determined. Blue vertical bar shows the Wellington score after pooling data without shuffling. Green: Wellington footprint score in fibroblast data. Red: footprint score in CD19 data. As pooling without shuffling yields a better footprint score than pooling with shuffling the footprint is considered non-differential.

**Figure S4. Example of de novo motif discovery in differential footprints**. HOMER de novo motif discovery results in footprints differentially enriched in CD19 versus CD4 lymphocytes. The top 6 motifs are shown sorted by increasing p-value.
